# “Magnitude of community-based health insurance utilization and associated factors in Bassona Worena District, North Shoa Zone, Ethiopia: a community-based cross-sectional study”

**DOI:** 10.1186/s12913-022-08794-6

**Published:** 2022-11-24

**Authors:** Tomas Getahun, Lakech Teklesilassie, Mizan Habtemichael, Yonas Abebe, Helen Getahun

**Affiliations:** 1 Armauer Hansen Research Institute - Ministry of Health, Addis Ababa, Ethiopia; 2 Department of Public Health, College of Health Sciences, Debre Berhan University, Debre Birhan, Ethiopia; 3 College of Health Sciences, Addis Ababa University, Addis Ababa, Ethiopia; 4 Department of Nursing, College of Health Sciences, Debre Berhan University, Debre Birhan, Ethiopia

**Keywords:** Community-based health insurance, Ethiopia, Health insurance, North Shewa Zone, Utilization

## Abstract

**Introduction:**

The health insurance system has been proven to offer effective and efficient health care for the community, particularly community-based health insurance is expected to ensure health care access for people with low economic status and vulnerable groups. Despite the significance of evidence-based systems and implementation, there is a limited report about the magnitude of CBHI utilization. Therefore, this study was done to assess factors associated with community-based health insurance utilization in Basona Worena District, North Shewa Zone, Ethiopia.

**Method:**

A community-based cross-sectional study was employed. We have included 530 households from 6 randomly selected kebeles. The data was entered using Epi-Data V 3.1 and exported to SPSS version 20.0 for statistical analysis. Bi-variable and multivariable logistic regression analyses were computed to determine factors associated with community-based health insurance utilization.

**Result:**

The study finding shows that 58.6% of the respondents were members of community-based health insurance. Respondents who had primary and secondary education levels were 2 times more likely to be members than those who had no formal education. As compared to those who had awareness, respondents who had no awareness about CBHI were 0.27 times less likely to be insured. Respondents who did not experience illness were 0.27 times less likely to be members than respondents who experienced illness.

**Conclusion:**

Educational status, awareness about CBHI, perception of CBHI scheme and illness experience of family influence CBHI utilization. There is a need to strengthen awareness creation to improve the CBHI utilization.

## Introduction

According to the World Health Organization (WHO), despite the wide variation in per capita health expenditure between and within countries of the world, over 100 million people are driven into poverty because of the catastrophic health spending. It is conceivable that the majority live in most middle-income countries and low-income countries (LMIC) which have poor health service system and weak health insurance scheme [1, 2].

This financial gap in LMIC leads to user fees for the health services they get that result in high out of pocket (OOP) expenditure. The high OOP expenditure because of user fees result financial risk and increases lack of fairness in access to essential health services [3]. High OOP payment for essential health services is the main risk factor of poverty that shows the need for improving economic security to minimize high health expenditure and reduce poverty [4]. In most LMIC, only a few people have access to health insurance scheme, peoples face health expenditure catastrophe because of absence of health financial risk protection [5].

In Ethiopia, the sources of health care financing are government, OOP, donors and other sources like health insurance [6]. Majority (more than 80%) of finance source in private health institution is OOP at the time of service delivery that indicates the poor health system structure of private health care expenditure. It is anticipated that CBHI scheme will strengthen the health system and help to stabilize the health care financing [7].

Health insurance provides financial risk protection and reduces poverty by risk pooling and it has also good possibility of ensuring universal health care coverage. Yet, launching such type of scheme in resource limited countries has been found challenging due to the exclusion of poor individuals in these areas and the majority are informal sectors employee and rural resident [3, 4]. Thus community based health insurance (CBHI) initiated in response to financial risk protection in health service and it is promising alternative for risk pooling health care financing system improves health services utilization and reduces poverty [8].

CBHI scheme has been implemented in developing countries and other parts of world. Recently in Ethiopia two type of health insurance scheme introduced which are social health insurance and CBHI. In social health insurance enroll the formal sector while CBHI involves informal sector of the economy. The social health insurance and CBHI scheme differ by premium collection system and the economy sector they enroll. CBHI system ensure the financial risk protection of poor individuals from health catastrophic expenditure through risk pooling and sharing and help health institutions to efficiently utilize resources and reduce financial risk [9].

WHO review report regarding health insurance enrollment of informal employee people shows that the utilization of non-profit health insurance system is very poor and only few members are enrolled [10]. There is also evidence that the implementation of CBHI in most LMICs continued to be challenged by poor enrollment of the community and sustainability concerns [11].

For instance, a community based cross-sectional study in South India that assessed determinants of CBHI utilization rural population reported 53.1% of the study participants were insured [12]. From African countries, the implementation of CBHI scheme ranged from about 2% in Kenya and Cameroon to 31% and 44% in Nigeria and Uganda respectively [13]. According to a cross-sectional study that was conducted to assess determinants of rural households willingness to participate in CBHI in Nigeria founds that only 31% of the study respondents were members of CBHI [14]. On another study done on determinants of CBHI enrollment and renewing among households in rural South-Western Uganda, they have found out that around 44% of the study respondents were member of CBHI scheme [15].

In Western Ethiopia, a cross-sectional descriptive study was conducted on CBHI utilization and its associated factors among informal workers in GIda Ayanan Ditrict, Oromia Region and it has found that founds that only 27.5% of the study participants enrolled in CBHI [16]. Another similar study conducted in West Gojjam Zone, Northwest Ethiopia revealed that 58.0% the study respondents were CBHI members [17]. Similar finding reported from studies conducted Dimibitchu and Damboya districts which found 67.0% and 62.0% respectively [9].

In Ethiopia, regardless of the benefit of evidence based study for policy and decision makers, there is limited amount of studies that are done on magnitude of CBHI utilization at district and country level [18]. To the best of the author’s knowledge, there is also no study or comprehensive studies that were conducted in Bassona Worenea district, North Shewa Zone, Ethiopia. Therefore, the current study aimed to determine the utilization of CBHI and its associated factors in Bassona Worenna District.Findings and recommendations from this study may provide information to policy makers and other concerned stakeholders at different level to design targeted and evidence based strategies that will help to increase the number of people utilize CBHI and it will also serve as a baseline for future studies in the area.

## Methods

### Study setting

This study was done in Bassona Worana district which is one of 24 districts in North Shoa Zone, Amhara Region, Ethiopia. The district is located in Northern part of Ethiopia and at 130 km distance far from Addis Ababa. It has large population size in North Shoa Zone with population number of 145,281 (in 2016). The district covers 786.5 km² areas and there are 31 kebeles and 35 health facilities.

#### Study design and period

A community based cross sectional study design was employed from June 1 to 30, 2020.

#### Sample size and sampling procedure

For this study, The actual sample was calculated using Epi-info statistical software by taking the proportion of CBHI utilization which is 94% from the national survey in Northwest Gojjam zone, Northwest Ethiopia. By considering design effect of two and 10% non-response rate a total of 530 representative households were selected. From the total 31 kebeles that are found in the district, six kebeles were selected using lottery method so Wayu, Debele, Saria, Nas, Kasima and Adisge were selected. The total sample size was allocated proportionally to each selected kebeles based on the number of households. Systematic random sampling technique was used to select study every 9th households using the administrative kebele household register.

#### Eligibility criteria

Permanent resident in the district ( for more than six months), household head aged 18 years old and above were qualified to be included in this study. Whereas, household heads who are a formal sector employees and who are unable to communicate due to being sick or other reasons were not included in the study.

#### Study variables

In this study CBHI utilization was our dependent variable and CBHI utilizers are operationalized as a households who are a member of community based health insurance who are confirmed by their new membership card.

whereas Socio-demographic factors such as age, sex, religion, ethnicity, marital status, educational level and monthly income, individual factors like awareness and perception about CBHI, health system related factors like distance from health facility, waiting time, availability of drugs, satisfaction with services and household’s health status were our independent variables.

#### Data collection tools and procedures

A structured questionnaire was developed based on the objectives of the study after reviewing previous literatures. The questionnaire includes information about respondent socio-demographic characteristics, their awareness and perception regarding CBHI, and questions about health-related factors. Four data collectors (health extension workers) and two supervisors (clinical nurses) were trained about the aim of the study the actual data collection.

#### Data quality control

The questionnaire was first prepared in the English language, then it is translated to the local language and to check the consistency of translation, the language expert translated it back to English. To check the uniformity of responses and understandability of the questions, the questionnaire was pre-tested in randomly selected 41 households at DebreSina District, North Shoa zone. Principal investigator and supervisors daily supervised the well trained data collection process, and they checked for completeness and uniformity of responses.

#### Data management and analysis

Data was entered using EPi-data version 3.3 and exported into SPSS version 20.0 for analysis. Different frequency tables, graphs and descriptive summaries were used to describe the study variables.

Bivariable logistic regression analysis was used to see significance of association between dependent and independent variable. In the bivariable analysis if the *p*-value ˂ 0.20 it was transferred to the multivariable logistic regression analysis, which help to control confounders. Odds ratioand 95% confidence interval are computed to measure the strength of the association between the outcome and the explanatory variables. P-value ˂0.05 considered as a statistical significant.

## Results

### Socio-demographic characteristics

In this study, 503 respondents took part with a 95.0% response rate. Around, 65.0% (327) respondents were male and 40.4% (203) of respondents were in the age group of 30 to 39 years while the median age being 35 years old. The majorities (85.7% (431)) of respondents’ religious belief were Orthodox and 66.4% (334) were Amhara in their ethnic group. Around 76.1% (383) of respondents were married and 40.0% (201) had no formal education. Regarding monthly income, 239 (47.5%) households earned over 2000 birr per month (Table 1).

**Table 1:** Socio-demographic characteristics of respondents, BassonaWorenaWoreda, Ethiopia 2020 (*n*= 503)

Characteristics	Frequency	Percent (%)
Sex		
Male	327	65.0
Female	176	35.0
Age in years		
Less than 30	154	30.6
30 to 39	203	40.4
40 and above	146	29.0
Religion		
Orthodox	431	85.7
Protestant	58	11.5
Muslim	9	1.8
Catholic	5	1.0
Ethnic Group		
Amhara	334	66.4
Oromo	151	30.0
Tigrey	18	3.6
Marital Status		
Single	53	10.5
Married	383	76.1
Divorced	29	5.8
Separated	21	4.2
Windowed	17	3.4
Educational Level		
No formal Education	201	40.0
Primary and secondary	131	26.0
College diploma and above	171	34.0
Monthly income		
Less than 1000 birr	153	30.4
1000 to 2000 birr	111	22.1
Above 2000 birr	239	47.5

Concerning the health status of the respondents and their family members, 46.7% (235) of them said their family health status was good and 2.4% (12) believed their family has very poor health status. Around 45.3% (228) said as their family fallen ill in the last six months.

### Respondent’s awareness and perception on community based health insurance

Regarding respondent awareness about CBHI, 86.5% (435) of them heard about CBHI and they are classified as aware group. Respondents who ever heard about CBHI scheme were asked about their source of information and 15.3% (77) of them heard about CBHI from neighbors or friends, 55.9% (281) heard from CBHI officials.

Concerning perception towards CBHI scheme, only 37.5% (352) of respondents said CBHI enrollment should not be only for those who are sick. Around 72.2% (363) of them agreed only very poor who cannot afford to pay for health care need to join the CBHI scheme. About 44.6% (362) respondents agreed under CBHI program, people pay money to finance future health care needs (Table 2).

**Table 2:** Respondent’s perception about CBHI, BassonaWorenaWoreda, Ethiopia 2020

Perception items	Frequency	Percent (%)
Only those who fall sick should consider enrollment in CBHI		
Correct	46	9.0
Not correct	352	37.5
Don’t know	105	33.5
Only the very poor who cannot afford to pay for health care need to join the schemes		
Correct	34	6.8
Not correct	363	72.2
Don’t know	106	21.1
Under CBHI program, you pay money (premiums) in order for the CBHI to finance your future health care needs?		
Correct	362	44.6
Not correct	37	27.4
Don’t know	104	28.0
CBHI program are like savings scheme, you will receive interest and get your money back		
Correct	49	9.7
Not correct	348	69.2
Don’t know	106	21.1
Do you think that the CBHI benefit package meets the requirements of you household?		
Yes	416	82.7
No	87	17.3
Do you think that CBHI management is trust worthy?		
Agree	395	78.5
Do not know	89	17.7
Disagree	19	3.8

### CBHI utilization

Among the total 503 respondents, 58.6% (295) of them reported as they were member of CBHI scheme. From the insured respondents 70.2% (207) visited health facility for illness. Among those who didn’t visit health facility about 58.0% (51) of them said it was not necessary. Among respondent who visited health facility for illness felt in the last six months, about half 49.3% of them were satisfied by the services given, 49.3% (102) of respondents said the waiting time to get services were less than thirty minutes and 37.2% (77) of them replied as drugs were usually available in the health facility, around 71.5% (211) of CBHI members used the scheme to cover the cost for the health services they got. For 65.5% (55) of the respondents, the major reason for not being a CBHI member was that no household member has visited health facilities which were reported by.

From the total insured CBHI members, 20.3% (60) mentioned that they will not renew their membership and absence of household member that has visited health facilities were a reason mentioned by 60.0% (36) of the respondents. Around 16.7% (10) of them said its due to limited availability and poor quality of health services, 13.3% (8) of them assumed that the quality of service for CBHI members is not as good as for non-CBHI members and 10.0% (6) of them said the registration fee and premiums are not affordable (Table 3).

**Table 3:** Respondents CBHI utilization, BassonaWorenaWoreda, Ethiopia 2020

	Frequency	Percent (%)
Currently member of CBHI scheme		
Yes	295	58.6
No	208	41.4
Visited the contractual health facility for the illness felt in the last 6 month		
Yes	207	70.2
No	88	29.8
How satisfied is with the services given?		
Very satisfied	50	24.2
Satisfied	102	49.3
Indifferent	41	19.8
Dissatisfied	11	5.3
Very dissatisfied	3	1.4
Waiting time to get the services		
Less than 30 minutes	102	49.3
30 to 60 minutes	56	27.1
More than 60 minutes	69	23.7
Availability of drugs in health facility		
Not available	15	7.2
Rarely available	39	18.8
Usually available	77	37.2
Always available	76	36.7
Major reason to did not visit a health facility		
Did not feel necessary	51	57.9
Facility too far	19	21.6
Lack of money for transportation	6	6.8
Did not feel I would get quality care	12	13.7
Use of your CBHI membership to cover health costs		
Yes	211	71.5
No	84	28.5
Why has your household not benefitted from CBHI?		
No one in my HH has visited health facilities	55	65.5
We still pay other additional costs for treatment (specify)	13	15.5
The quality of service for CBHI members is not as good as for non-CBHI members	7	8.3
Delays in issuance and distribution of CBHI ID cards	9	10.7
Renewal of CBHI membership for the following year		
Yes	235	79.7
No	60	20.3
Why do you plan not to renew your CBHI membership?		
Illness and injury does not occur frequently in our HH	36	60.0
The registration fee and premiums are not affordable	6	10.0
There is limited availability and poor quality of health services	10	16.7
The quality of service for CBHI members is not as good as for non-CBHI members	8	13.3

### Factors associated with community based health insurance utilization

In bivariate analysis, variables that showed significant association with CBHI utilization with *p* value < 0.2 were included in the multiple logistic regression analysis (Table 4). Based on the analysis output, socio-demographic characteristics (such as age, sex, religion, ethnicity, marital status, educational level and monthly income,) and awareness about CBHI, perception towards CBHI, health status and illness experiences of family were significantly associated with CBHI utilization.

**Table 4:** Factors associated with CBHI utilization, BassonaWorenaWoreda, Ethiopia 2020 (n= 503)

Characteristics	CBHI utilization		COR (95% CI)	AOR (95% CI)
	Yes	No		
Educational Level				
No formal Education	105 (52.2)	96 (47.8)	1	1
Primary and secondary	98 (74.8)	33 (25.2)	2.715 (1.1677, 4.396)^a^	2.336 (1.168, 4.673) ^a^
College diploma and above	92 (53.8)	79 (46.2)	1.065 (0.708, 1.602)	0.819 (0.424, 1.581)
Awareness about CBHI				
Yes	281 (64.6)	154 (35.4)	1	1
No	14 (20.6)	54 (79.4)	0.142 (0.076, 0.264) ^a^	0.270 (0.113, 0.648) ^a^
CBHI benefit package meets the requirements of your household				
Yes	278 (66.8)	138 (33.2)	1	1
No	17 (19.5)	70 (80.5)	0.121 (0.068, 0.213) ^a^	0.188 (0.085, 0.415) ^a^
Health status of you and your family				
Very good	60 (43.8)	77 (56.2)	1	1
Good	149 (63.4)	86 (36.6)	2.223 (1.447, 3.416)	2.329 (1.376, 3.943) ^a^
Acceptable	44 (62.0)	27 (38.0)	2.091 (1.164, 3.758) ^a^	1.509 (0.714, 3.191)
Poor	32 (66.7)	16 (33.3)	2.567 (1.289, 5.110) ^a^	2.407 (0.910, 6.367)
Very poor	10 (83.3)	2 (16.7)	6.417 (1.55, 30.391) ^a^	2.500 (0.447, 1.998)
Have you and your family fallen ill in the last 6 months?				
Yes	174 (76.3)	54 (23.7)	1	1
No	121 (44.0)	154 (56.0)	0.244 (0.166, 0.359) ^a^	0.272.164, 0.451) ^a^

^a^ significantly associated with *p* value < 0.05

In multivariable logistic regression analysis level of educational, awareness about CBHI, perception towards CBHI and illness experience of family showed a significant association with CBHI utilization. Respondents who had primary and secondary educational level were 2 times more likely to be CBHI member than those who didn’t have formal education (AOR = 2.336; 95% CI=: 1.168, 4.673). Respondents who had no awareness about CBHI were 0.27 times less likely to become CBHI members compared to those who had awareness about the scheme (AOR = 0.270; 95% CI=: 0.113, 0.648). Likewise, respondents who do not perceived CBHI scheme meets their household requirement were 0.188 times less likely to utilize CBHI than those who had perceived the CBHI benefit package meets their household requirement (AOR = 0.188; 95% CI=: 00.085, 0.412). Furthermore, utilization of CBHI among respondents who did not experience illness was 0.27 times less likely to utilize CBHI than who experienced illness in the last six months (AOR = 0.272; 95% CI=: 0.164, 0.451).

## Discussion

The main purpose of this study was to assess CBHI utilization in BassonaWorena Woreda, North Shoa Zone, Ethiopia. This study also identified factors that associated with CBHI utilization. The result of this study revealed that only 58.6% of respondents were CBHI member. This finding is in line with study conducted in West Gojjam Zone, Northwest Ethiopia in which 58.0% of the study respondents were CBHI members [17]. But, different from a descriptive study in Gida Ayanan Ditrict, Oromia Region, West Ethiopia (27.5%) [16]. on a study done on determinants of CBHI enrollment and renewing among households in rural Southwestern Uganda (44%) [15]. The possible cause of the discrepancy could be due to the variation in access to information regarding CBHI.

In this study one of factors that affected CBHI utilization was respondent educational level. This study found out that respondents who had primary and secondary educational level were 2 times more likely to be CBHI member than those who hadn’t joined a formal education. Similar finding reported from study conducted in South India where higher education showed statistically significant positive association with CBHI scheme utilization [19]. Several studies also revealed that educational status was factor that showed statistically significant association with decision to enroll in CBHI [20, 21].  Individuals who have higher educational level might know the importance and method of risk sharing in CBHI which can lead them to become a member [16].

Majority (86.5) of this study respondent had awareness about CBHI. This result is higher than CBHI utilization reported from study that examined dropping out of Ethiopians CBHI scheme where 69% of CBHI uptake found [22]. The difference seen could be due to extensive awareness creation campaign done by CBHI officials in recent time. The result of this study further showed that respondents who ever heard about CBHI scheme were asked about their source of information and majority, 55.9% of them heard from CBHI officials.

In addition, this study finding showed that respondents who had no awareness about CBHI were less likely to be CBHI member compared to those who had awareness about the scheme. Similarly, a community based cross-sectional study conducted in Kenya reported that awareness about CBHI scheme had significant association with decision to enroll in CBHI [23]. The possible explanation might be knowing the benefits of enrolling in the CBHI scheme changes their health seeking practice [16].

Positive perception and understanding the CBHI benefit packages facilitate utilization of the scheme. In this study respondents who do not perceived CBHI scheme meets their household requirement were less likely to utilize CBHI than those who had perceived the CBHI benefit package meets their household requirement. Study done in Edo state of Nigeria revealed that perception of CBHI scheme meets household requirement had negative association with CBHI utilization [24].

Even though, no particular illness specified in this study respondents who had history of familiar illness in the last six months were more likely to be CBHI member. Nevertheless, respondents who did not experience illness were less likely to be CBHI member. This result is similar with study conducted in Cameroon which reported family with history of illness had more tendencies to uptake CBHI scheme [25]. This could be due to if there is familiar history of illness there might be higher health care expenses that increases the likelihood to enroll in CBHI.

From the total insured CBHI members, about 20.3% of them would not renew their membership for the following year and their reason to did not renew their membership were no one in my HH has visited health facilities, limited availability and poor quality of health services and the quality of service for CBHI members is not as good as for non-CBHI members. This result is supported by study conducted in Burkina Faso [26].

## Conclusion and recommendation

This study found that even though most of respondents have awareness about CBHI, the utilization of is low. Educational status, awareness about CBHI, perception towards CBHI scheme and illness experience of family, influence CBHI utilization. Furthermore, limited availability of drugs, poor quality of services and the quality of the services for CBHI members are not as good as for non-member are the important reasons for CBHI member to do not renew their membership in the following year. In order to improve the CBHI utilization, there should be a strong monitoring evaluation system to ensure the quality of services and availability of drugs for CBHI members, local level information dissemination regarding the benefit of CBHI scheme, the amount of premium paid and the period of payment for renewal should be strengthened. This study can be used as baseline information for further studies on this topic to explore reasons for low utilization of CBHI scheme by assessing challenges and opportunities.

## Data Availability

The datasets generated during the current study are not publicly available due to participants have not agreed to make this dataset publically available. But it will be available from the corresponding author on reasonable request after making adjustments to hide anonymity of the participants.

## Abbreviations

AOR: Adjusted Odds Ratio
CBHI: Community Based Health Insurance
COR: Crude Odds Ratio
OOP: Out of Pocket
LMIC: Low and Middle Income Countries
SPSS: Statistical Package for the Social Sciences
WHO: World Health Organization
